# A Simple Method for On-Gel Detection of Myrosinase Activity

**DOI:** 10.3390/molecules23092204

**Published:** 2018-08-31

**Authors:** Sándor Gonda, Zsolt Szűcs, Tamás Plaszkó, Zoltán Cziáky, Attila Kiss-Szikszai, Gábor Vasas, Márta M-Hamvas

**Affiliations:** 1 Department of Botany, Division of Pharmacognosy, University of Debrecen, Egyetem tér 1, H-4010 Debrecen, Hungary; vapenda@gmail.com (Z.S.); plaszkotomi@gmail.com (T.P.); vasas.gabor@science.unideb.hu (G.V.); hamvas.marta@science.unideb.hu (M.M.-H.); 2 Agricultural and Molecular Research and Service Institute, University of Nyíregyháza, Sóstói str. 31/b, H-4400 Nyíregyháza, Hungary; cziaky.zoltan@nye.hu; 3 Department of Organic Chemistry, University of Debrecen, Egyetem tér 1, H-4010 Debrecen, Hungary; attyska@gmail.com

**Keywords:** myrosinase, thioglucosidase, sulfatase, on-gel detection, desulfo-sinigrin, LC-ESI-MS

## Abstract

Myrosinase is an enzyme present in many functional foods and spices, particularly in Cruciferous vegetables. It hydrolyses glucosinolates which thereafter rearrange into bioactive volatile constituents (isothiocyanates, nitriles). We aimed to develop a simple reversible method for on-gel detection of myrosinase. Reagent composition and application parameters for native PAGE and SDS-PAGE gels were optimized. The proposed method was successfully applied to detect myrosinase (or sulfatase) on-gel: the detection solution contains methyl red which gives intensive red bands where the HSO_4_^−^ is enzymatically released from the glucosinolates. Subsequently, myrosinase was successfully distinguished from sulfatase by incubating gel bands in a derivatization solution and examination by LC-ESI-MS: myrosinase produced allyl isothiocyanate (detected in conjugate form) while desulfo-sinigrin was released by sulfatase, as expected. After separation of 80 µg protein of crude extracts of Cruciferous vegetables, intensive color develops within 10 min. On-gel detection was found to be linear between 0.031–0.25 U (pure *Sinapis alba* myrosinase, *R*^2^ = 0.997). The method was successfully applied to detection of myrosinase isoenzymes from horseradish, Cruciferous vegetables and endophytic fungi of horseradish as well. The method was shown to be very simple, rapid and efficient. It enables detection and partial characterization of glucosinolate decomposing enzymes without protein purification.

## 1. Introduction

The glucosinolate-myrosinase-isothiocyanate system is a widely distributed chemical defense system of the Brassicales [1]. As the volatile isothiocyanates are highly bioactive molecules that are at the same time beneficial to human consumers, the system is of high scientific and industrial interest [2].

**Figure 1:**
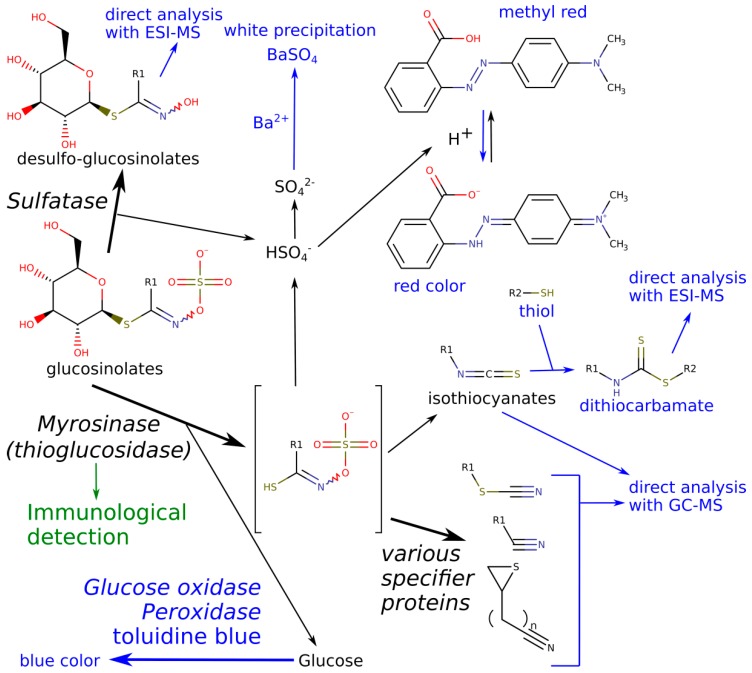
The main breakdown scheme of glucosinolates with possible detection methods of the products. Enzymatic reactions are shown with bold arrows, enzymes have italic font. The reactions that occur in vivo, are shown in black. Detection methods are either blue (those which require an operating myrosinase) or green (methods reliant only on similarity of sequences). The default rearrangement products of the thiohydroximates are isothiocyanates, alternative reaction products (from top to bottom) are nitriles, thiocyanates and epithionitriles. In our model, R1 = allyl- (sinigrin is converted to allyl isothiocyanate), R2 = 2-aminoethyl- (cysteamine).

The plants biosynthesize the glucosinolate precursors, which come in contact with their activation enzyme myrosinase under some circumstances, usually when tissue damage occurs [1]. The reaction catalyzed by myrosinase (EC 3.2.1.147) is a thioglucoside hydrolysis which results in an unstable thiohydroximate that subsequently undergoes spontaneous rearrangement (Figure 1). The default products are isothiocyanates, or, in vivo in the presence of so called specifier proteins, other less toxic volatiles can be formed. The activity itself is shown to be present in various Brassicaceae plants [3,4], microorganisms [5,6,7], and organisms associated with such plants, like endophytes from horseradish roots [8] or some insects feeding on host plants with such metabolites [9,10,11]. A simple plant can contain various myrosinase isoenzymes, as shown in *Arabidopsis thaliana* [12] and other Brassicaceae plants [13,14,15,16].

Full characterization of enzymes requires purification during which activity is usually monitored by a routine, specific assay. Purification of enzymes with this activity was successful from several sources, including various Brassicaceae plants [4,13,17,18], microorganisms [6,7] or insects [11,19]. Myrosinase assays usually detect either the decomposition of the substrate (glucosinolates), or release of one of the product compounds. The possibilities include direct detection of volatile products by GC-MS [20,21], measurement of the released acid (pH stat assay) [22], a decrease of concentration of the glucosinolate substrate via spectrophotometry or chromatographic techniques [23,24], or derivatization of the released glucose (Figure 1) via coupled enzyme reactions [25]. The detection of the enzyme by immunological methods [26,27,28] is also a viable option.

Detection by immunological methods are very specific, and monoclonal anti-myrosinase antibodies, such as 3D7 are available for research [13,27,28]. However, while they can also detect myrosinases in inactive (denaturated) form, they are unable to detect proteins that have myrosinase activity, but are structurally unrelated to that used for antibody production. As they have evolved independently, myrosinases from different sources can vary considerably, which is perhaps highlighted by the fact that ascorbic acid inhibits the myrosinases of a cabbage aphid [11], marginally activates the myrosinase of *Citrobacter* [6], and activates plant myrosinases to a high extent [13]. If a myrosinase is to be detected that has unrelated peptide sequence to that of the known ones, insufficient binding of the primary antibody is likely.

Unfortunately, the above activity-based methods interfere with later protein purification steps, or suffer from other limitations. On-gel detection methods of myrosinases (thioglucosidases) include that of [29,30,31] who used Ba^2+^ to form a whitish precipitate from the released SO_4_^2−^. This approach also worked when screening fungal isolates for such an activity [32]. However, there are also limitations. Ba^2+^ forms white precipitates with a variety of anions (phosphate, carbonate, citrate), detection and documentation of the pale white color in the transparent gels can be a challenge, especially when low amounts of myrosinase is present. Perhaps therefore, there are no exact sensitivity data presented for on-gel usage in the literature. Ba^2+^ can also strongly bind to some proteins (see e.g., [33]). Though no data is available on this phenomenon with special respect to myrosinases, it might interfere with subsequent purification and characterization. Another disadvantage is that Ba^2+^ is highly toxic and requires special disposal. Another approach to detect myrosinase activity was the method of [34] who used starch gels, and used the glucose-oxidase-peroxidase-*o*-toluidine mixture, which results in blue colors if free glucose is present. This method has the advantage that it is more specific for myrosinase (sulfatase does not liberate any glucose, Figure 1), but the obtained gel sample is not suitable for later purification or characterization because of the added enzymes.

Seeing the limitations of the above, we aimed to develop a sensitive, straightforward on-gel assay for myrosinase that can also be used to detect bands on native PAGE gels or SDS-PAGE gels, after a simple washout protocol. Also, the development of an LC-ESI-MS method capable of distinguishing myrosinase from sulfatase was aimed.

## 2. Results and Discussion

### 2.1. On-Gel Detection of Glucosinolate Decomposition

Our approach was to detect the release of H^+^, a side product of the myrosinase catalyzed glucosinolate hydrolysis (Figure 1). The detection was planned to be accomplished using a pH indicator in a weakly buffered reaction mixture. As myrosinase operates over a wide pH range [13], the produced acidification, detected by the pH indicator, does not inactivate the enzyme.

Preliminary tests to choose the proper pH indicator were done in test tubes, using crude extracts of horseradish roots containing high amounts of myrosinase. Congo red, bromocresol green and methyl red were selected for the test, as they show color transition within the range pH 4–6. Methyl red was chosen for further work as it provided the most spectacular color change during the in-vial assay, its color transition from yellow to red can be observed in the pH range 6–4.4. After addition of 10 µL myrosinase containing horseradish crude extract (typical protein content 45 µg) to 90 µL of the unbuffered detection reagent, the mixture developed intensive reddish color usually within a few minutes.

The least buffering capacity that still resulted in a stable solution (i.e., no spontaneous acidification and color change within 24 h) was found to be 1 mM phosphate, pH 7.5, methyl red concentration was 100 µg mL^−1^. This was supplemented with the amount of sinigrin (6 mM) and ascorbic acid (1 mM) usually used in on-gel detection assays [31]. Hence, the final composition of the detection reagent was 6 mM sinigrin, 1 mM ascorbate, 1 mM Na_2_HPO_4_, pH 7.5, 100 µg mL^−1^ methyl red.

The detection reagent was successfully used “as is” for on-gel detection. After washing of native gels containing separated proteins of horseradish crude extracts, the myrosinase containing bands were successfully detected using the proposed detection reagent. Many enzymes can release acid, but the reaction conditions made the assay specific to glucosinolate decomposition: the proposed detection reagent does not produce color change in the absence of sinigrin as shown in a PAGE of horseradish crude extracts (Figure S1).

**Figure 2:**
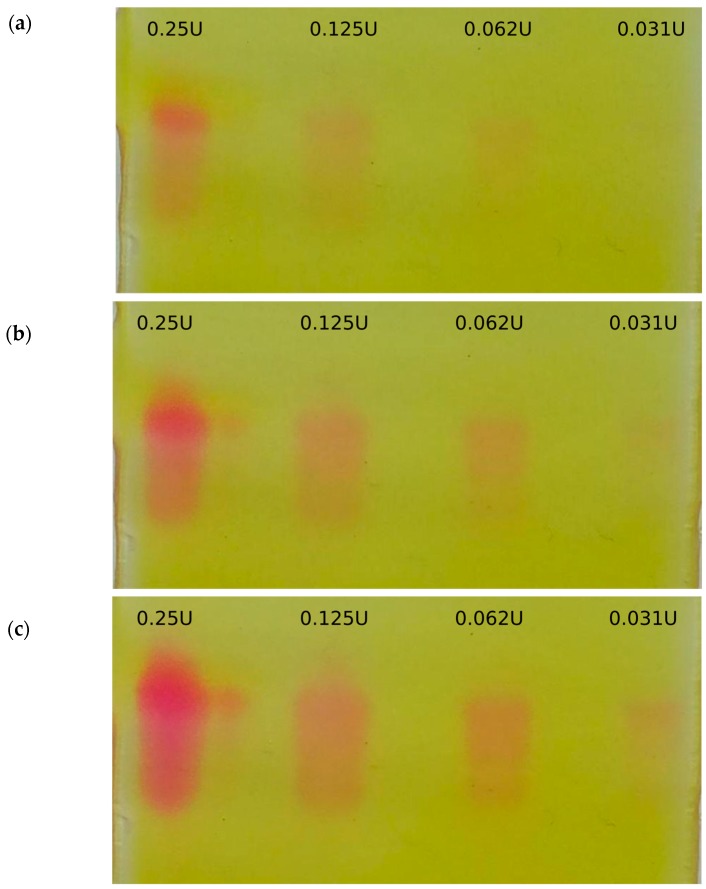
Short-term time course of the detectable on-gel signal—a serial dilution of myrosinase standard was detected with the proposed detection reagent containing sinigrin, 6 mM; ascorbic acid, 1 mM; Na_2_HPO_4_, 1 mM; pH 7.5; methyl red, 100 µg mL^−1^. 0.031–0.25 enzyme units (U) of *Sinapis alba* thioglucosidase (myrosinase) standard) was separated on 7.5% native PAGE. Subplots: (**a**) 4 min, (**b**) 6 min, (**c**) 8 min.

The reaction was also positive with purified myrosinase. The bands from purified myrosinase from *Sinapis alba* seeds (Sigma Aldrich, St. Louis, MO, USA) at 0.031–0.25 U, developed colors within 8 min (Figure 2, Figure S2c and Figure S3). The gel in Figure 2 was photographed at 4, 6 and 8 min and evaluated by CP Atlas 2.0 gel image processing software (green channel). At 0.125 and 0.25 U, linear relationship was found between the signal and the incubation time (*R*^2^ ≥ 0.996). At 4 min, *R*^2^ = 0.997 signal—activity linearity was obtained in the range 0.031–0.25 U (also see Table S1). This means that besides qualitative detection, approximate activity data can be obtained using the proposed method, within the given activity range. It is worth to note, that in case of extremely low activities, it was possible to left the gel covered for hours to detect minute amounts of myrosinase: no spontaneous acidification (red background increase) was observed in such gels, supporting the stability of the mixture in the absence of enzymes. The same amount of myrosinase did not result any white bands of BaSO_4_ precipitation after the attempt to detect with the detection reagent of [31].

**Figure 3:**
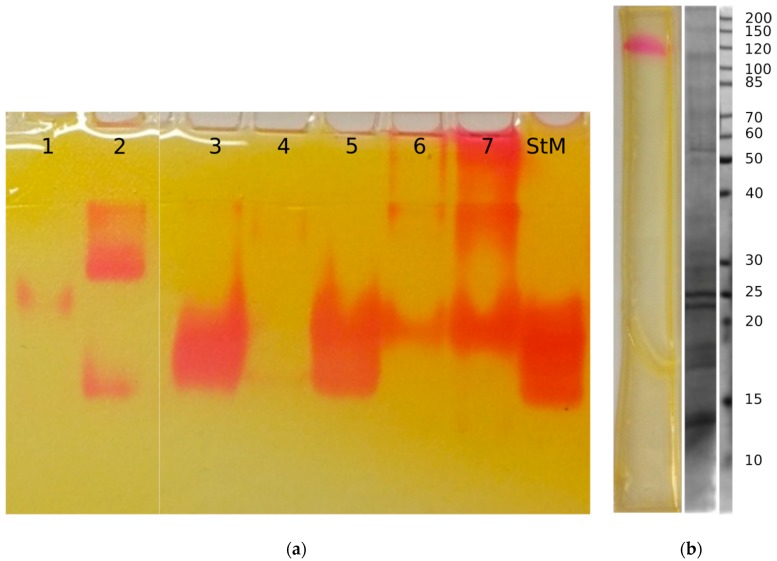
On-gel detection reaction of myrosinases from crude extracts of different species of Brassicaceae. Subplots: (**a**) Crude extracts with 80 µg protein content were loaded on 7.5% native PAGE. Samples: 1: *Brassica oleracea var. gemmifera* buds; 2: *Brassica oleracea var. italica* flowering heads, 3: rocket salad (*Eruca sativa*) whole seedlings, 4: *Brassica oleracea var. botrytis* flowering heads, 5: *Sinapis alba* whole seedlings, 6–7: *Armoracia rusticana* roots from two different sources, StM: *Sinapis alba* myrosinase standard. (**b**) 10% SDS-PAGE of horseradish root (*Armoracia rusticana*, 80 µg total protein) crude extracts. Myrosinase activity was detected by the proposed detection reagent after wash-out of SDS (**left**). The protein pattern of the horseradish root crude extract (**center**), and the molecular weight marker (Page RulerTM Unstained Protein Ladder, Thermo Scientific, **right**) were stained with Coomassie-Brillant Blue.

The *S. alba* thioglucosidase as well as other plant myrosinase enzymes retained their activity after separation on SDS-PAGE gels and washout of SDS (Figure 3b, Figure S2b,c and Figure S3, Table S2). At 4 min, *R*^2^ = 0.9899 signal—activity was also observed (Table S2). The proposed washout procedure ensures elimination of the high amount of buffer and SDS that is typical during gel electrophoresis.

If desired, a higher sensitivity can be reached (at the cost of lower stability) by using a detection reagent with a slightly lower pH, this results in earlier color development (Figure S4).

### 2.2. LC-ESI-MS of Products of Separated Sulfatase and Myrosinase Enzymes

As sulfatase also releases H^+^ and SO_4_^2−^ from sinigrin (Figure 1), a distinction has to be made whenever there is a possibility that sulfatase is present instead of myrosinase. This was carried out by LC-ESI-MS. We expected that incubation of sinigrin with myrosinase results in the release of allyl isothiocyanate (AITC) while sulfatase produces desulfosinigrin (Figure 1). LC-ESI-MS has the ability to detect desulfosinigrin with high sensitivity and specificity, while ITCs are usually detected by GC-MS, however, we wanted a single distinguishing measurement. Therefore, given our experience with ITC derivatization with thiols [24], we tested several thiols for ITC derivatization and LC-MS detection.

Several thiols with various side-chains that can be expected to ionize well under ESI-MS conditions were purchased and tested to react with 1 µg mL^−1^ allyl isothiocyanate in 50 mM NH_4_OAc buffer (pH 9.0). The amount of ITC-thiol adduct (dithiocarbamate) produced is a function of pH [24], which is controlled by addition of the buffering agent at 40-fold excess compared to the reagent.

After testing several derivatization reagents, cysteamine was found to form the most sensitively detectable ITC derivates (Figure 1). The calibration curve in LC-ESI-MS was linear in the ranges 100–10^5^ ng mL^−1^ for the cysteamine derivate (AITC equivalent, injecting 1 µL of derivatized sample). The LOD for cysteamine–AITC adduct was as low as 1 ng mL^−1^. As compared to other LC-ESI-MS methods, this LOD is in the range of the most sensitive ITC determination methods [35,36]. MS/MS characterization of the cysteamine adduct (*m*/*z* 177.051) showed a major characteristic fragment 160.0248 ([M − NH_3_ + H]^+^, calcd. 160.0249, difference 0.6 ppm) as well as less intensive fragments *m*/*z* 101.0423 and 73.0112.

After optimization of the derivatization method and the LC-ESI-MS parameters, sulfatase standard (*Helix pomatia*), myrosinase standard (*Sinapis alba*) and horseradish crude extract were separated on slab gels and detected using the proposed detection reagent. The *Sinapis alba* myrosinase and horseradish crude extract yielded two and three bands, respectively, while sulfatase standard contained only one. Thereafter, the active bands were cut out and incubated in a derivatization solution containing 0.24 mM sinigrin, 100 mM NaH_2_PO_4_ buffer (final pH 7.5), 1 mM ascorbic acid and 46.1 mM cysteamine. Our previous study has shown that small aliphatic thiols are compatible with the myrosinase reaction, no enzyme inhibition was observed with mercaptoacetic acid in the reaction medium [24].

As expected, reaction with the band containing sulfatase resulted in production of desulfo-sinigrin, while reaction with all of the myrosinase bands (three from horseradish or two from *Sinapis alba*) resulted in allyl isothiocyanate which subsequently spontaneously conjugated with cysteamine (Figure 1 and Figure S5). Desulfosinigrin was identified using literature data [37]. The monoisotopic exact mass of the [M + H]^+^ obtained was 280.0846 which matched the calculated value of 280.0849 (difference: −1.1 ppm). In MS/MS, the thioglycoside loses the glucose moiety, leading to a characteristic fragment C_4_H_8_NOS at *m*/*z* 118.0323 (calculated 118.0321, difference: −1.7 ppm). In case of the allyl-isothiocyanate adduct, the previously detected dithiocarbamate product was formed and detected by LC-ESI-MS at *m*/*z* 177.051. Hence, sulfatase and myrosinase were successfully distinguished using LC-ESI-MS without addition of any substance or enzyme that might hinder possible later protein purification or characterization steps.

### 2.3. On-Gel Detection after Separation of Crude Extracts

The main applications of the given detection method is to aid myrosinase purifications, and to study glucosinolate decomposing enzyme pattern of different organisms, which was achieved successfully using the proposed procedure: a series of crude extracts from different Brassicaceae plants were separated and their glucosinolate decomposing enzymes detected (Figure 3a). It is apparent, that most vegetables contain different complexes or isoenzymes that show the activity of interest. In case of the three isoenzymes from horseradish root crude extract, all three isoenzymes were proven to be myrosinases by detecting their reaction product allyl-isothiocyanate by LC-ESI-MS. As sulfatase activity is not described from Brassicaceae plants so far, the active bands of other vegetables most likely myrosinase isoenzymes or different complexes thereof. Perhaps because of the heavy glycosylation of these enzymes, separation efficacy in native PAGE and SDS-PAGE were similar (Figure S3a,b). In horseradish samples separated on SDS-PAGE, the MW of the myrosinase band approximately matches that published by [13] (Figure 3b).

Successful detection of glucosinolate-decomposing enzymes was also achieved from two of the endophytic fungi of horseradish, *Fusarium oxysporum* and *Macrophomina phaseolina*, which were recently shown to possess decomposing activity towards plant glucosinolates [8] (Figure S6). As allyl-isothiocyanate-GSH conjugate was detected from the spent medium, but desulfoglucosinolates were not [8], these organisms also likely possess myrosinase activity. The crude extract of the fungi (prepared with the same methodology as the crude plant extract) were shown to be orders of magnitude less active than that of the vegetables.

## 3. Materials and Methods

### 3.1. Chemicals

All reagents were of analytical purity. Allyl isothiocyanate and cysteamine were from Sigma-Aldrich (St. Louis, MO, USA). Congo red, bromocresol green and methyl red were from Reanal (Budapest, Hungary). Sinigrin was from Phytoplan (Heidelberg, Germany). Buffer components (disodium phosphate, ammonia, acetic acid, ammonium acetate) and ascorbic acid were from VWR (Debrecen, Hungary). As water, type I water (18.2 MΩ cm^−1^) was used, which was produced by a Human Zeneer Power I water purification system (Human corporation, Seoul, Republic of Korea).

### 3.2. Pure Enzymes

Myrosinase (thioglucosidase) standard from *Sinapis alba* was from Sigma Aldrich, 1 U is equivalent to 1.0 μmole glucose min^−1^ from sinigrin at pH 6.0 at 25 °C. The used lot showed 260 U g^−1^ activity. Sulfatase standard from *Helix pomatia* was from Sigma Aldrich, 1 U was equivalent to hydrolysis of 1.0 μmole *p*-nitrocatechol sulfate h^−1^ at pH 5.0 at 37 °C (30 min assay). The enzyme used showed 30,320 U g^−1^ activity.

### 3.3. Organisms

Wild or commercial samples of the following plants were used: horseradish (*Armoracia rusticana*) root; *Armoracia macrocarpa* leaf; white mustard (*Sinapis alba*) seeds and whole seedlings; black mustard (*Sinapis nigrum*) seeds, black radish *(Raphanus sativus* var. *sativus*) root, rocket salad (*Eruca sativa*) leaf and seedlings, Brussels sprouts (*Brassica oleracea* var. *gemmifera*) buds, broccoli (*Brassica oleracea* var. *italica*) flowering heads and cauliflower (*Brassica oleracea* var. *botrytis*) flowering heads.

Selected horseradish endophytes from our previous study [8] (*Fusarium oxysporum*, *Macrophomina phaseolina*) were grown in inactivated horseradish extract until reaching stationary phase of growth in which decomposition of sinigrin usually initiates (5 and 7 days, respectively). The inactivated horseradish extract was prepared using boiling methanol, therefore it does not contain any active enzymes. It was standardized to contain 12 mM (5 mg mL^−1^) sinigrin. The preparation of the inactivated horseradish extract and the inoculation parameters were the same as in our recent work [8].

### 3.4. Sample Preparation

The enzyme containing crude extracts were prepared as in our recent study [24]. Briefly, the raw plant material was mixed in a blender with cold 25 mM phosphate buffer (pH 6.5), centrifuged (13,000 rpm, 5 min), filtered on PES 0.22 μm membrane if necessary, and used directly for gel electrophoresis after protein content determination by a Bradford assay [38] as described earlier.

### 3.5. Testing of Detection Reagents

Congo red, bromocresol green or methyl red was dissolved in MeOH (1 mg mL^−1^), and added to a mixture containing 6 mM sinigrin, 1 mM ascorbic acid, pH 8.0. To a 90 µL aliquot of the above mixture, 10 µL of horseradish crude extract (equivalent to about 45 µg protein) was added. The reaction mixture was subsequently incubated for about 15–30 min. As a positive control, 10 µL 0.1 M HCl was added to the mixture. To negative controls, 10 µL distilled water was added.

The final solution consisted of 1 mM Na_2_HPO_4_, 1mM ascorbic acid, 100 µg mL^−1^ methyl red (from a 1 mg mL^−1^ solution of methyl red in MeOH), 6 mM (2.5 mg mL^−1^) sinigrin. The pH of the solution was adjusted to 8.0 with 0.1 M NaOH solution before adding methyl red and sinigrin. Addition of the residual components decreased the pH to a final value of 7.5.

### 3.6. Gel-Electrophoresis

Polyacrylamide slab gels (5.7% top/stacking and 10 or 7.5% bottom/resolving gels) with discontinuous buffer system were prepared according to [31,39] and used for on-gel detection of myrosinase. The optimal protein content of samples loaded onto gels were 20–200 µg depending on plant samples. After electrophoresis at 4 °C in dark, 20–22 mA/gel, gels were washed several times in purified/distilled water at room temperature to wash out the buffering electrolytes. For the successful reaction, the measured pH of the spent washing water was comparable to that of the washing distilled water (6.85–7.4). In our setup, samples were allowed to overrun for 35 min which made the separation of the more slowly migrating proteins better.

10 and 7.5% SDS-PAGE were used to investigate the ability of myrosinases for renaturation. Plant crude extracts were loaded together with molecular weight marker (PageRuler Unstained Protein Ladder 200–10 kDa, BLUeye prestained Protein Ladder 245–11 kDa, Sigma-Aldrich) to estimate molecular weight. After electrophoresis, renaturation of proteins was performed by washing out SDS in 20% (*v*/*v*) 2-propanol in water (2 × 15 min.). These steps were followed by washing with sterilized distilled water to obtain the optimal pH.

### 3.7. On-Gel Detection

For the reaction, 4 mL detection reagent was uniformly distributed on the surface of 90 × 120 mm gel on a glass or plastic foil. When samples with very different myrosinase activities are investigated, using of striped slab gels is recommended to avoid the cross-contamination between lanes. The typical reaction time of our plant samples was 10 min.

### 3.8. LC-ESI-MS

The UHPLC system (Ultimate 3000RS, Dionex, Sunnyvale, CA, USA) was coupled to a Thermo Q Exactive Orbitrap mass spectrometer (Thermo Fisher Scientific Inc., Waltham, MA, USA) equipped with an electrospray ionization source (ESI). The column was a Phenomenex Kinetex XB-C_18_ column (100 mm × 2.1 mm × 2.6 μm), oven temperature was maintained at 30 °C, flow rate was 250 μL min^−1^. Eluent A was 100% water and eluent B was 100% acetonitrile. Both contained 0.1% formic acid as modifier. The following gradient elution program was used: 0 min, 95% A, 0–2 min, 95% A; 2–5 min, 75% A; 5–6 min, 40% A; 6–7 min, 0% A; 7–9 min, 0% A; 9–10 min, 95% A, 10–18 min, 95% A. 1 μL of the samples were injected in every run. The Q Exactive hybrid quadrupole-orbitrap mass spectrometer was operated in positive ion mode with the following parameters: capillary temperature 320 °C, spray voltage 4.0 kV, the resolution was set to 70,000. The mass range scanned was 150–1000 *m*/*z*. The maximum injection time was 100 ms. The resolution was set to 17,500 in the cases of MS^2^ scans. The collision energy was 30 NCE. Sheath gas and aux gas flow rates were 32 and 7 arb, respectively.

### 3.9. Enzyme Reaction for Distinguishment of Myrosinase and Sulfatase

2.5 U of sulfatase and myrosinase standard as well as horseradish 80 µg protein was separated on a native gel. The gel pieces showing activity with the proposed reagent were washed with water and subsequently immersed in the derivatization solution, and incubated overnight at room temperature. The derivatization solution consisted of 3.56 mg mL^−1^ (46.1 mM) cysteamine, 100 mM NaH_2_PO_4_, 1 mM ascorbic acid and 240 mM (100 µg mL^−1^) sinigrin. The pH of the solution was adjusted to 7.7 with 10M NaOH solution. The solution was diluted 25-fold with MeOH, centrifuged and 1 µL was injected to LC-ESI-MS as described above.

## 4. Conclusions

An inexpensive and simple method has been developed for on-gel detection of glucosinolate-decomposing enzymes, which is based on release of the HSO_4_^−^, i.e., the acidification of the reaction medium which is detected by methyl red pH indicator. When sufficient enzyme is present (which is the case when loading 80 µg protein for most tested Brassicaceae vegetables) the reaction is rapid (color develops within 10 min). Subsequent analysis by LC-MS allows one to distinguish myrosinase from sulfatase activity when necessary. The proposed detection method enables rapid detection of myrosinases and sulfatases from different sources after on-gel separation. We think the method can be of use for food chemistry research, functional characterization of the plant microbiome, chemical ecologists, and plant physiology studies as well.

## Supplementary Materials

The supplementary are available on line.
