# Supplementary material for: A Simple Method for On-Gel Detection of Myrosinase Activity
Source: Molecules. 2018 Aug 31;23(9):2204. doi: 10.3390/molecules23092204 (PMC6225493; doi:10.3390/molecules23092204)
Supplement: Supplementary file 1 [file molecules-23-02204-s001.pdf]

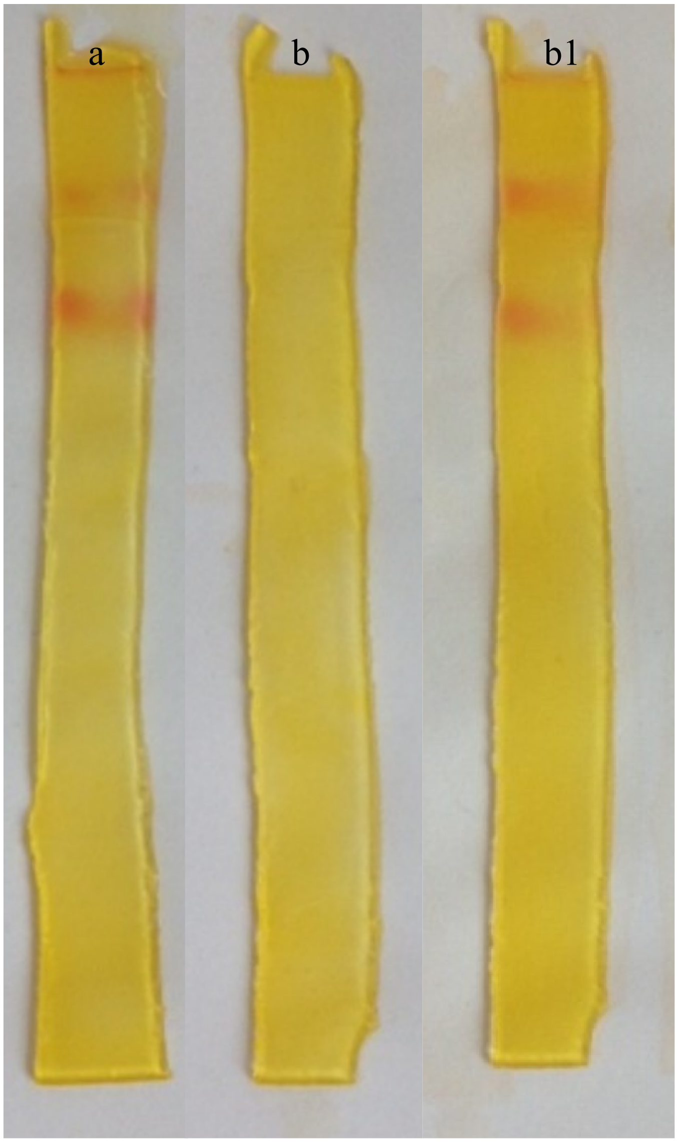

Figure S1. Selectivity of the proposed detection reaction. Myrosinase detection from 40 µl of horseradish root crude extract (approximately 60 µg protein) separated on 7.5% native polyacrilamide gel. After electrophoresis, gels were agitated in sterilized distilled water to wash out the electrolytes. Subplots:

a: On-gel detection of myrosinase by the proposed detection reagent: positive reaction after 8 minutes.

b: On-gel detection attempt of acid-releasing enzymes by the proposed detection reagent **without the myrosinase substrate sinigrin**: no reaction.

b1: On-gel detection by the proposed detection reagent on band "b" after it failed to show any sinigrin-independent signal: positive reaction after 8 minutes.

Proposed detection reagent: sinigrin, 6 mM; ascorbic acid 1 mM; Na_2HPO_4, 1mM; pH 7.5; methyl red 100 µg mL^{-1}.

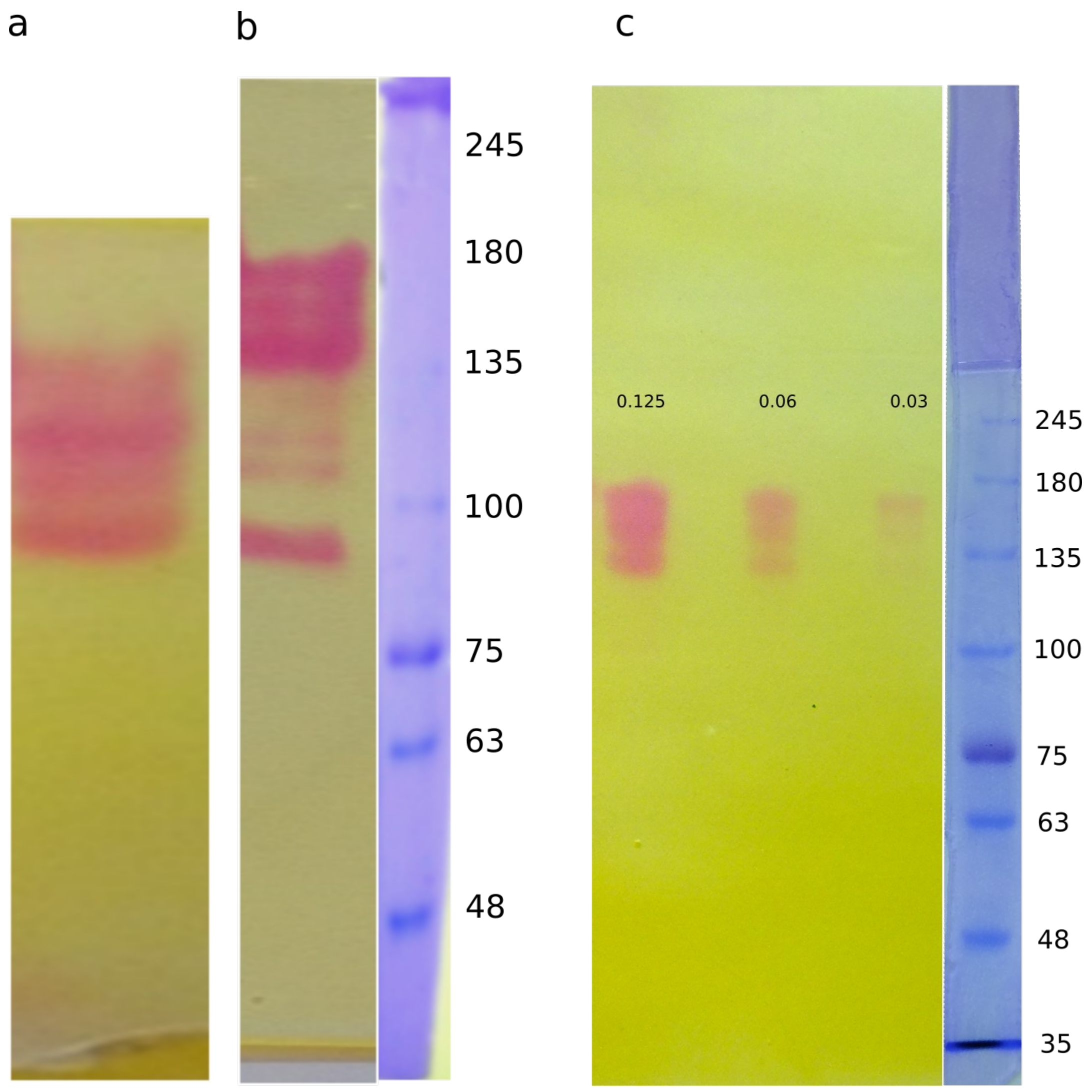

Figure S2. Myrosinases of *Sinapis alba*, separated and detected with the proposed procedure. High load is because of the high amount of storage proteins (subplots a, b).
Subplots: a., Separation of crude extract of *S. alba* seeds (200 µg protein) on 7.5% native gel; b., Separation of crude extract of *S. alba* seeds (200 µg protein) on SDS-PAGE with 3% SDS in sample loading buffer. BLUeye Prestained Protein Ladder was used as the molecular marker. c., Myorinase standard from *Sinapis alba* (Sigma Aldrich) separated on SDS-PAGE (0.125, 0.06 and 0.03 U, as indicated). BLUeye Prestained Protein Ladder was used as the molecular marker.

a

b

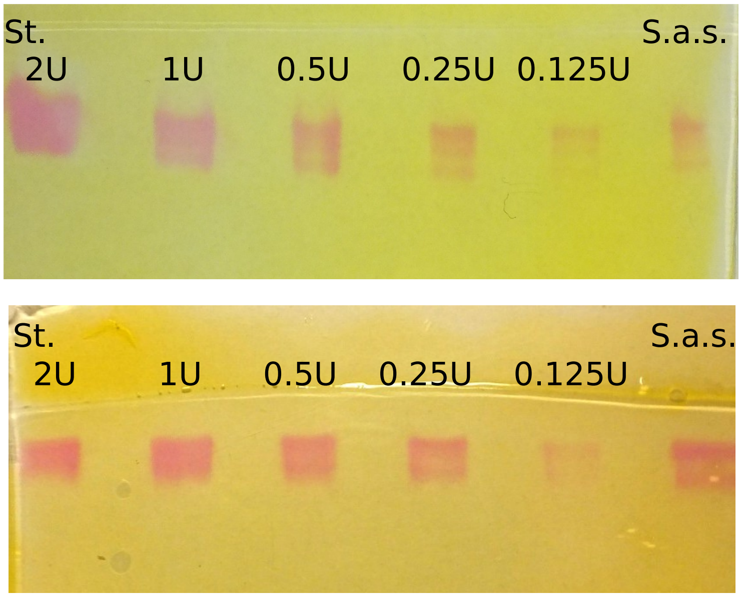

Figure S3. A serial dilution of purified *Sinapis alba* myrosinase and a crude extract of the same plant detected with the proposed reagent, after separation on a., native PAGE, b., 10% SDS-PAGE (after washout). Samples: St.: 0.125 - 2 U of *Sinapis alba* thioglucosidase (myrosinase) standard; S.a.s.: crude extract of 3-day-old *Sinapis alba* seedlings (80 µg total protein content).

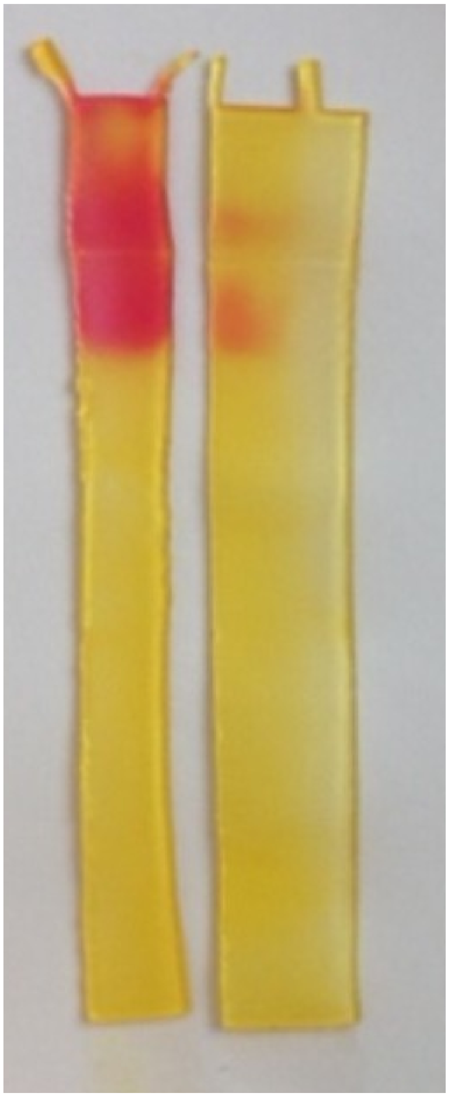

Figure S4.
Effect of pH during detection of myrosinases from *A. rusticana* (horseradish) samples separated on native PAGE gels and detected by the proposed reagent. Left: pH 6.4, right: pH 7.2.

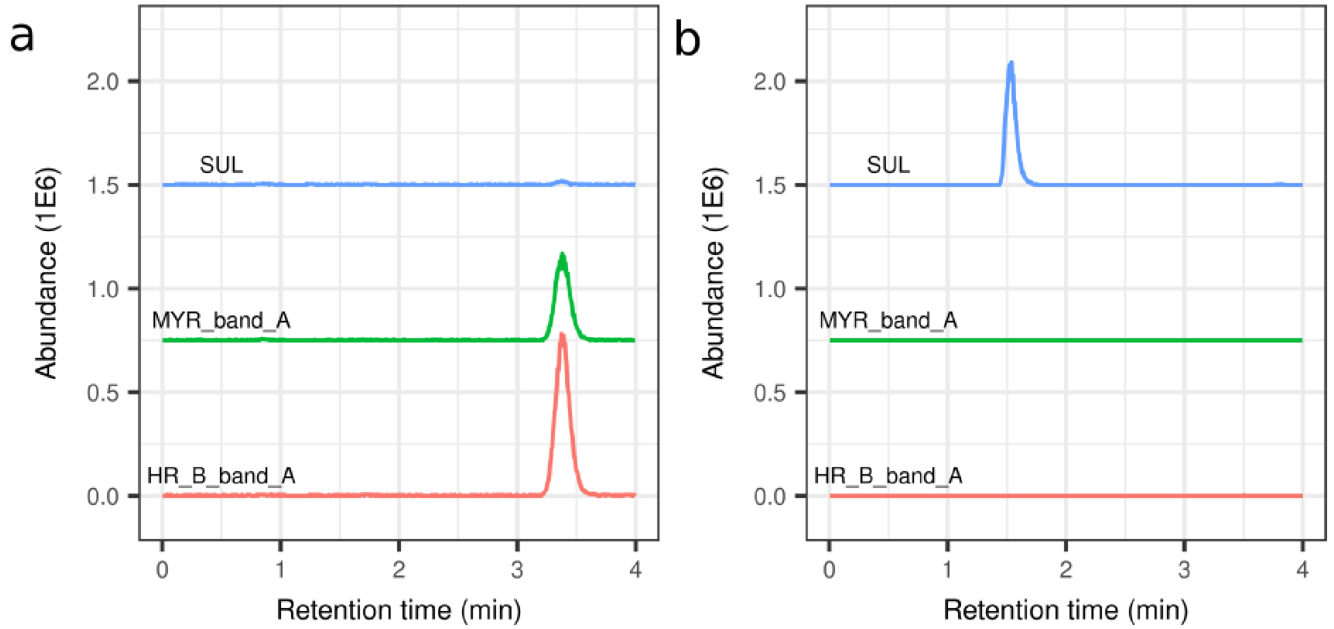

Figure S5. LC-ESI-MS chromatograms of cutout gel bands incubated with sinigrin in the presence of cysteamine for identification of sinigrin decomposition products and distinguishment of sulfatase activity from myrosinase activity. Subplots: a., Extracted ion chromatograms of m/z range of 177.0515 ± 5 ppm, showing production of allyl isothiocyanate, detected in the form of cysteamine conjugate at retention time 3.2 min. b., Extracted ion chromatograms of m/z range of 280.0849 ± 5 ppm, showing production of desulfo-sinigrin at retention time 1.55 min. All samples were separated on PAGE gels before the cutout of the band. Samples: MYR_band_A, a band from *Sinapis alba* thioglucosidase standard; HR_B_band_A, a band from *Armoracia rusticana* (horseradish) crude extract; SUL, the band from sulfatase standard (*Helix pomatia*).

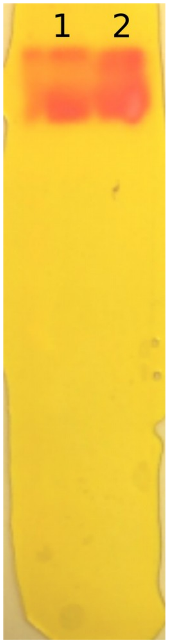

Figure S6.
Crude extracts of endophytic fungi containing myrosinase enzymes as detected with the proposed reagent, after separation on 10% SDS-PAGE and washout. Incubation time was 2 hours. Samples: 1: *Fusarium oxysporum*, endophyte from horseradish roots; 2: *Macrophomina phaseolina*, endophyte from horseradish roots.

Table S1.

Raw area under curve data and linearity data for Fig. 2. (on-gel detection of a serial dilution of *Sinapis alba* myrosinase on native gel) using the proposed detection procedure after image processing by CP Atlas 2.0 software.

U (μmol min^{-1}) \\ Time	4 min	6 min	8 min	R^2 (Time)
0.25 U	4878	9011	13498	0.9994
0.125 U	2408	3741	5637	0.9900
0.0615 U	1024	2168	3857	0.9878
0.031 U	211	681	1361	0.9890
R^2 (enzyme amount)	0.9976	0.9912	0.9828	

Table S2.

Raw area under curve data and linearity data for Fig. S2c. (on-gel detection of a serial dilution of *Sinapis alba* myrosinase on SDS-PAGE) using the proposed detection procedure after image processing by CP Atlas 2.0 software.

U (μmol min^{-1}) \\ Time	4 min	6 min	8 min	R^2 (Time)
0.25 U	3827	6155	11795	0.9455
0.125 U	1947	3693	7544	0.9550
0.0615 U	450	1236	2966	0.9552
0.031 U	143	306	950	0.8941
R^2 (enzyme amount)	0.9899	0.9741	0.9643	
